# Mathematical Description of Bacterial Traveling Pulses

**DOI:** 10.1371/journal.pcbi.1000890

**Published:** 2010-08-19

**Authors:** Jonathan Saragosti, Vincent Calvez, Nikolaos Bournaveas, Axel Buguin, Pascal Silberzan, Benoît Perthame

**Affiliations:** 1 Physico-Chimie-Curie, Institut Curie, UMR CNRS 168, Paris, France; 2 Unité de Mathématiques Pures et Appliquées, École Normale Supérieure de Lyon, UMR CNRS 5669, Lyon, France; 3 NUMED, INRIA Rhône-Alpes, Montbonnot, Lyon, France; 4 School of Mathematics, University of Edinburgh, Edinburgh, United Kingdom; 5 Laboratoire Jacques-Louis Lions, Université Pierre et Marie Curie, UMR CNRS 7598, Paris, France; 6 Institut Universitaire de France, Paris, France; University of Illinois at Urbana-Champaign, United States of America

## Abstract

The Keller-Segel system has been widely proposed as a model for bacterial waves driven by chemotactic processes. Current experiments on *Escherichia coli* have shown the precise structure of traveling pulses. We present here an alternative mathematical description of traveling pulses at the macroscopic scale. This modeling task is complemented with numerical simulations in accordance with the experimental observations. Our model is derived from an accurate kinetic description of the mesoscopic run-and-tumble process performed by bacteria. This can account for recent experimental observations with *E. coli*. Qualitative agreements include the asymmetry of the pulse and transition in the collective behaviour (clustered motion versus dispersion). In addition, we can capture quantitatively the traveling speed of the pulse as well as its characteristic length. This work opens several experimental and theoretical perspectives since coefficients at the macroscopic level are derived from considerations at the cellular scale. For instance, the particular response of a single cell to chemical cues turns out to have a strong effect on collective motion. Furthermore, the bottom-up scaling allows us to perform preliminary mathematical analysis and write efficient numerical schemes. This model is intended as a predictive tool for the investigation of bacterial collective motion.

## Author Summary

Modeling chemotaxis has raised a lot of interest in the applied mathematics community in past decades. The precise description of bacterial pulses traveling in a narrow channel is a challenging issue in the self-organization of cells. Indeed, our biological knowledge of signal integration in *E. coli* has grown in parallel with the development of more involved mathematical models. There exists a hierarchy of models for the analysis of bacteria *E. coli* motion depending on the scale under consideration. In this work, we derive macroscopic equations from the mesoscopic scale. This allows us to perform qualitative and quantitative analysis based on numerical simulations. We compare our predictions with current experiments performed with *E. coli*. The results can be interpreted at the cellular scale due to the bottom-up integration. This approach reveals better agreement with current experiments than the widely used Keller-Segel model. We conclude that the mesoscopic run-and-tumble description is compatible with the propagation of a pulse at the macroscopic scale.

## Introduction

Since Adler's seminal paper [1], several groups have reported the formation and the propagation of concentration waves in bacteria suspensions [2], [3]. Typically, a suspension of swimming bacteria such as *E. coli* self-concentrates in regions where the environment is slightly different such as the entry ports of the chamber (more exposed to oxygen) or regions of different temperatures. After their formation, these high concentration regions propagate along the channel, within the suspension. It is commonly admitted that chemotaxis (motion of cells directed by a chemical signal) is one of the key ingredients triggering the formation of these pulses. We refer to [4] for all biological aspects of *E. coli*.

**Figure 1:**
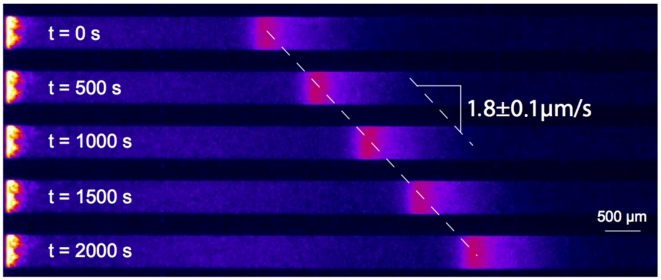
Experimental evidence for pulses of *Escherichia coli* traveling across a channel. The propagation speed is constant and the shape of the pulse front is remarkably well conserved. Observe that the profile is clearly asymmetric, being stiffer at the back of the front (see also Fig. 2). Cell division may not play a crucial role regarding the short time scale.

Our goal is to derive a macroscopic model for these chemotactic pulses based on a mesoscopic underlying description. This approach relies on kinetic theory adapted to the specific run-and-tumble process that bacteria undergo [5], [6]. We base our modeling task on recent experimental evidence for traveling pulses obtained in our group (Fig. 1). These traveling pulses possess the following features which we are able to recover analytically: constant speed, constant amount of cells and strong asymmetry in the profile.

Many other micro-organisms exhibit collective behaviors. For instance, *Dictyostelium discoideum* cells collectively switch their cAMP-mediated signaling activity from stochastic to oscillatory when a concentration threshold is reached [7]. These oscillations, then synchronized at the scale of the population, give rise to non-dissipating waves of cAMP that guide the cells during fruiting body formation. Another example is given by *Myxococcus xanthus* that can grab and pull a neighbor cell by the mean of their pili, resulting in a long range alignment of the population and the formation of aggregates [8]. In the first case the pulsatile aspect is crucial for population scale communication and the speed of the cAMP waves is one order of magnitude larger than the velocities of the individual cells. In the second case, the physical contact between cells is critical for the aggregation.

Mathematical models for chemotaxis highlight a positive feedback which counteracts dispersion of individuals and may eventually lead to aggregation. There is a large amount of literature dealing with this subtle mathematical phenomenon (cf. [9], [10] and the references therein, see also [11] for alternative models which are closer to our approach). Self-induced chemotaxis following the Keller-Segel model has been shown successful for modeling self-organization of various cell populations undergoing aggregation [12]–[15].

In particular the Keller-Segel model has been proposed as a basis for modeling the propagation of traveling waves [16]–[19]. We refer to [20] for a complete review of contributions to this modeling issue. It has been postulated that a single chemotactic signal, namely the nutrient, could be responsible for the motion of the wave. However it is required that the chemosensitivity function is singular when the nutrient concentration vanishes. Our approach is more robust as we give a large class of fluxes for which traveling pulses do propagate. Furthermore these fluxes are derived from an accurate mesoscopic description of bacterial interactions.

In addition to chemotaxis, the contribution of cell division has been considered by many authors (cf. [21]–[23] and the references therein). Following the theory of reaction-diffusion equations, these authors have demonstrated the existence of traveling waves under general assumptions. However taking into account population growth seems unreasonable in view of the time scale of the experimental setting we aim at describing.

An extension of the classical Keller-Segel model was also proposed in seminal paper by Brenner *et al.* [24] for the self-organization of *E. coli*. Production of the chemoattractant by the bacteria triggers consumption of an external field (namely the succinate). Their objective is to accurately describe aggregation of bacteria along rings or spots, as observed in earlier experiments by Budrene and Berg that were performed over the surface of gels [2]. However the experimental setting we are based on is quite different from Budrene and Berg's experiments: for the experiments discussed in the present paper, the bacteria swim in a liquid medium and not on agar plates. Therefore we will not follow [24]. On the other hand Salman et al. [25] consider an experimental setting very similar to ours. However the model they introduce to account for their observations is not expected to exhibit pulse waves (although the mathematical analysis would be more complex in its entire form than in [18]).

A new class of models for the collective motion of cells (*e.g.* swimming bacteria, the slime mold *D. discoideum*) has emerged recently. It differs significantly from the classical Keller-Segel model. Rather than following intuitive rules (or first order approximations), the chemotactic fluxes (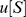, say: 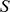 being the concentration of a chemotactic cue) are derived analytically from a mesoscopic description of the run-and-tumble dynamics at the individual level and possibly involving internal molecular pathways, [9], [26]–[34]. The upscaling limit which links the macroscopic flux 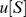 to the kinetic description is now well understood since the pioneering works [5], [6], [26]. Here we propose to follow the analysis in [11], [31]. We write accordingly the macroscopic chemotactic flux in full generality as:
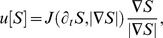
(1)where 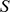 denotes the concentration of chemoattractant. We shall derive an explicit formulation for the macroscopic quantity 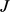. Indeed it contains the microscopic features that stem from the precise response of a single bacterium to a change in the concentration of the chemoattractant 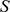 in its surrounding environment. The upscaling limit is based on the following experimental fact: the (collective) pulse speed and the (individual) speed of bacteria differ by one order of magnitude. To the best of our knowledge, this is the first work where this powerful approach has been applied to the propagation of bands in populations of *E. coli* [35].

## Results

### Description of the experiments

When confined in micro environment, motile populations of *Escherichia coli* exhibit robust collective behaviours in the form of propagation of concentration waves. If this phenomenon is relatively easy to observe, its quantitative study requires a reproducible preparation of the system. To do so, we perform the following experiment. Fluorescent bacteria are grown in a nutritive medium until they reach a sufficient density and a good motility. We then fill PDMS/glass micro channels directly with this suspension, or after resuspension in a different medium. The channels are then sealed with epoxy resin thus confining the homogeneous suspension of motile bacteria. The centrifugation of this system reproducibly accumulate bacteria at one end of the channel while preserving the motility. When the centrifugation is stopped, a sharp pulse forms and propagates along the channel. Fluorescence video microscopy allows the measurement of the speed and shape of the traveling pulse. Precise experimental details are given in material and methods.

### Description of the model

We describe the population of bacteria by its density 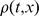 (at time 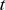 and position 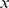). We consider here short timescales, hence cell division is assumed to be negligible. The cell density follows a drift-diffusion equation, combining brownian diffusion together with directed fluxes being the chemotactic contributions. This is coupled to reaction-diffusion equations driving the external chemical concentrations. In this paper we consider the influence of two chemical species, namely the chemoattractant signal 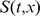, and the nutrient 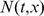. Although this is a very general framework, it has been shown in close but different conditions that glycine can play the role of the chemoattractant [25]. Similarly, glucose is presumed to be the nutrient. The exact nature of the chemical species has very little influence on our modeling process. In fact there is no need to know precisely the mechanisms of signal integration at this stage. The model reads as follows:
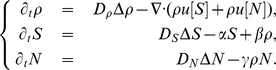
(2)


The chemoattractant is assumed to be secreted by the bacteria (at a constant rate 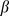), and is naturally degraded at rate 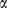, whereas the nutrient is consumed at rate 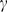. Both chemical species diffuse with possibly different molecular diffusion coefficients. We assume a linear integration of the signal at the microscopic scale, resulting in a summation of two independent contributions for the directed part of the motion expressed by the fluxes 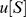 and 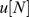. We expect that the flux 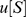 will contribute to gather the cell density and create a pulse. The flux 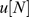 will be responsible for the motion of this pulse towards higher nutrient levels.

The fluxes 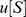 and 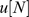 are built from the kinetic description of motion at the mesoscopic scale (see Materials and Methods). To summarize we assume that bacteria follow a run-and-tumble process mediated by the chemical micro-environment. The tumbling rate is dependent upon the material derivatives 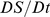 and 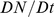 (see [11], [31] for related works), where 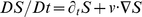, and 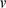 denotes the cell velocity. Namely, we assume that the tumbling rate writes as follows: 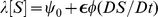. Here 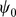 is the basal rate of tumbling in the absence of chemoattractant and 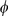 is a decreasing function: tumble is more likely to occur if the chemoattractant concentration decreases along the trajectory [36], [37]. The (small) parameter 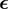 accounts for the small variations of tumbling rates which have been measured experimentally (results not shown). The synthesis of these phenonena yields a macroscopic equation for the cell density 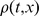 (2), where the chemical drift is given by
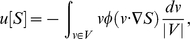
(3)where 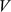 denotes the set of possible velocities. The same holds for 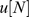. The dependency upon the time derivative 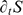 disappears due to time/space scaling. We could keep this dependency at first order, but we omit it for the sake of clarity.

Several systems such as (2) have been proposed and the upmost classical is the so-called Keller-Segel equation [12], [16]. In the latter, the fluxes are proportional to the gradient of the chemical: 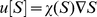, *resp.* 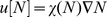. Such a coupling is known to possibly drive the system into aggregated configurations for which the density of cells can become unbounded [9]. Notice that the two possible choices coincide in the linear regime, *i.e.* for small amplitudes of 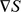. They strongly differ however far from the linear regime. Especially the flux 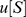 given by (3) is bounded by the individual speed of bacteria, whereas the chemotactic flux in the Keller-Segel model generally becomes unbounded when aggregative instability occurs, which is a strong obstacle to the existence of traveling pulses.

### Analytical solutions in the case of a stiff response function

We restrict our attention to the one-dimensional case due to the specific geometry of the channels. It is usually impossible to compute explicitely traveling pulse solutions for general systems such as (2). To obtain qualitative properties is also a difficult problem: we refer to [17], [18], [23] for examples of rigorous results in this direction.

Here, we are able to handle analytical computations in the limiting case where the signal response function 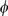 is indeed a step function. This owes to the assumption of high sensitivity of bacteria or large gradients of chemical. Then the fluxes (1) are given by the expression (3) which reduces to
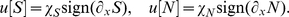
(4)


**Figure 2:**
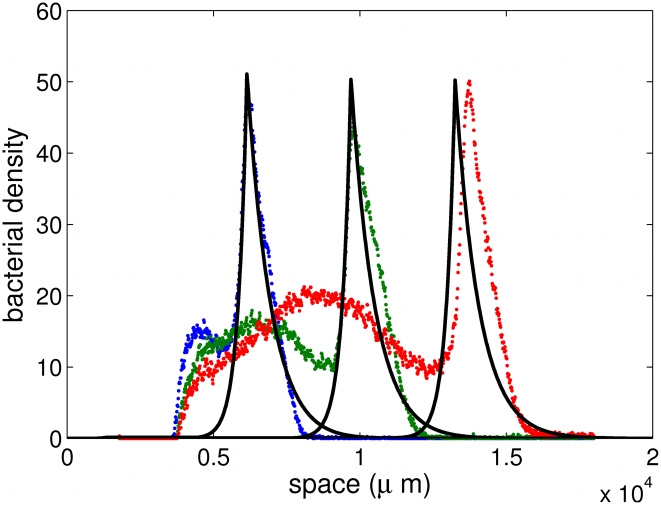
Comparison between experimental data and numerical results obtained from the model. Superposition of three time-snapshots of the experiments (*dots*, see also Fig. 1) and the numerical simulations of (2)–(4) (*plain line*). The time interval between snapshots is 2000*s*. The density profile is clearly asymmetric and preserved along the time course of the experiment. The number of bacteria in the pulse is approximately constant during the course (main contribution to growth takes place at the back of the pulse). The model reproduces faithfully the exponential tail at the back of the peak. The profile s do not coincide perfectly in the last snapshot due to uctuations in the experimental speed of propagation. Parameters chosen for the simulations are given in Table 1. The numerical speed is 1∶8µm.s^−1^.

We seek traveling pulses, in other words particular solutions of the form 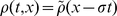, 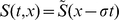, 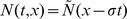 where 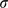 denotes the speed of the wave. This reduces (2) to a new system with a single variable 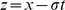,
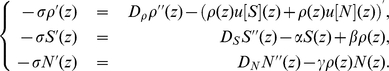
(5)We prescribe the following conditions at infinity
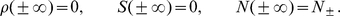
(6)We impose 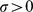 without loss of generality. This means that the fresh nutrient is located on the right side, and thus we look for an increasing nutrient concentration 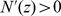. We expect that the chemoattractant profile exhibits a maximum coinciding with the cell density peak (say at 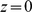), and we look for a solution where 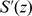 changes sign only once at 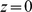. Then, the fluxes (4) express under the traveling wave ansatz as
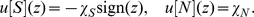
Integrating once the cell density equation in (5) we obtain
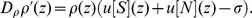
The flux 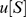 takes two values (with a jump at 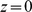), whereas the flux 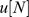 is constant. Therefore the cell density is a combination of two exponential distributions
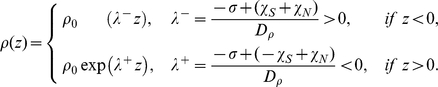
(7)This combination of two exponentials matches with the numerical simulations (Fig. 2) and the experimental observations (Fig. 2).

To close the analysis it remains to recover the two unknowns: the maximum cell density 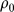 and the speed 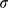, given the mass and the constraint that 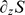 vanishes at 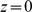 (because 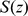 reaches a maximum at this location). We have the following implicit formula for the speed of the pulse (see Text S1 for details):
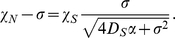
(8)We deduce from monotonicity arguments that there is a unique positive traveling speed 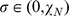.

On the other hand, the asymmetry factor is given by
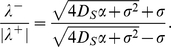
(9)This is a key macroscopic quantity as it enables to retrieve some parameters from experimental measurements. Interestingly enough, the speed and the asymmetry factor do not depend on the number of bacteria when the response function is stiff.

### Formation of bacterial clusters

Mittal *et al.* have presented remarkable experiments where bacteria *E. coli* self-organize in coherent aggregated structures due to chemotaxis [38]. The cluster diameters are shown essentially not to depend on the quantity of cells being trapped. This experimental observation can be recovered from direct numerical simulations of random walks [39].

We can recover this feature in our analytical context using a model similar to (2) derived from a kinetic description. We compute the solutions of (5) in the absence of nutrient (assuming again a stiff response function). Observe that stationary solutions correspond here to zero-speed traveling pulses, that is
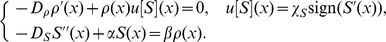
(10)We assume again that 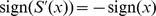. This simply leads to,
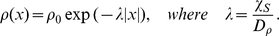
This is compatible with the postulate that 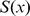 changes sign only once, at 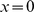 (the source 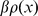 being even). The typical size of the clusters is of the order 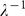, which does not depend on the total number of cells. This is in good quantitative agreement with experiments exhibited in [38]. The fact that we can recover them from numerical simulations indicates that these stationary states are expected to be stable.

Cluster formation provides a good framework for investigating pattern formation when we relax the stiffness assumption on the response function 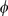. We introduce the stiffness parameter 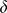 through its derivative at the transition between unfavourable and favourable regimes: 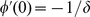. The case 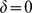 corresponds to a step response function.

We get from the dispersion relation (see Text S1) that the constant stationary state 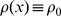 is linearly stable if and only if the following condition is fulfilled:
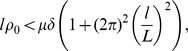
(11)where the constant 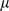 depends on the other parameters (including the mean square velocity 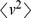, see Text S1). Here 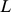 denotes the size of the channel and 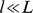 is the range of action of the chemical signal (namely 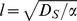). The picture is not complete as we have not investigated the stability of the non-trivial steady-state. However this indicates that the stiffness parameter 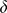 plays an important role regarding cluster formation. We show below that stiffness plays an important role for coherent motion of a pulse too.

### Numerical insights

**Figure 3:**
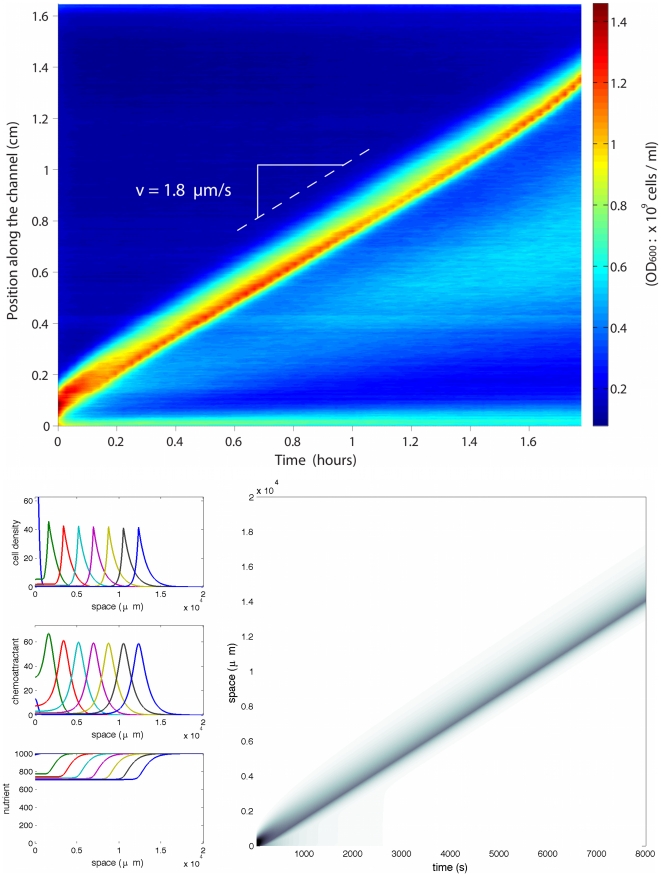
Propagation of a pulse wave. (Top) Experimental results under abundant nutrient conditions: M9 minimal medium supplemented with 4% glucose and 1% casamino acids (both ten times more concentrated than in the case of Fig. 5). (Bottom) Numerical simulations of system (2)–(3) in the case of unlimited nutrient, and a stiff response function *φ*. We observe the propagation of a traveling pulse with constant speed and asymmetric profile. Specific parameters are: (*δ* = 10^−1^ and *N*_0_ = 103 (arbitrary units).

We complete the theoretical analysis with some numerical simulations of the full model (2)–(3) exhibiting propagation of pulses (or not) in regimes where analytical solutions are not available (Fig. 3). The set of parameters is given in Table 1. The two parameters subject to variation are the stiffness parameter 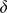 and the initial level of nutrient 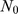.

**Table 1:** Set of parameters.

Time scale 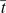	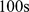	Experimental evidence
Space scale 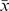	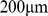	Experimental evidence
Effective bacterial diffusion 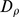	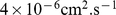	[47]
Chemical diffusion 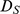	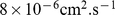	[4]
Chemical degradation 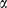	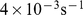	[25] and experimental fit
Effective bacterial chemotaxis speed 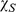	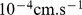	Experimental fit
Effective bacterial chemotaxis speed 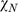	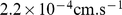	Experimental fit
*Response function* 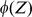	step or 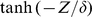	
*Total number of cells* 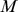	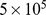	Experimental measurement
*Chemical secretion* 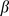	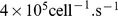	[25]
*Nutrient diffusion* 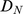	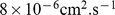	
*Nutrient consumption* 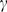	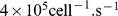	[25]

Reference set of parameters which have been used in the numerical simulations. The asterisks point out the parameters which have little influence on the dynamics: they have been chosen in agreement with the other parameters (with respect to the order of magnitude). The three parameters 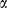, 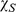 and 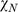 have been obtained from the macroscopic observables 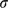, 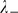 and 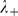 using the formulas (7), (8): 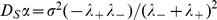, 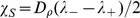, 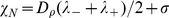. We obtain a value for 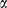 which is consistent with [25]. See Materials and Methods for details.

We can draw the following conclusions from our numerical simulations. The first remarkable fact is that we do observe traveling pulses (Fig. 3). Dispersion effects are counterbalanced by self-attraction due to the signal 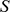. These traveling pulses possess the correct asymmetry in the profile, and the speed matches experimental observations.

**Figure 4:**
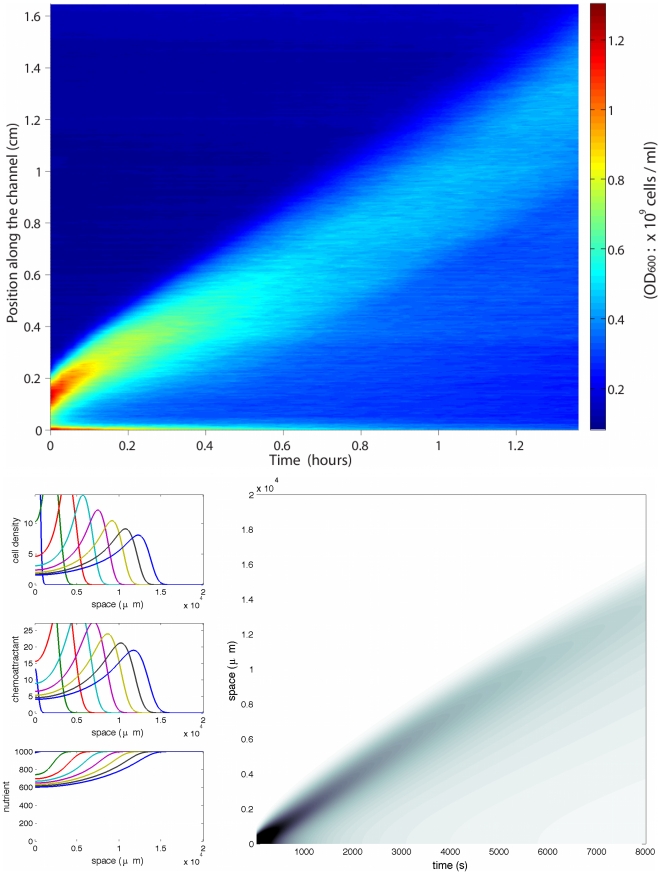
Dispersion of the cell population (no pulse wave). (Top) In this experiment, bacteria are cultivated at a concentration of 5.10^8^cells.ml^−1^ in the same rich medium as in Fig. 3. After, they are resuspended in LB nutrient to an OD600 of 3.10^8^cells.ml^−1^. We interpret the absence of pulse propagation as following. Bacteria are adapted to a rich environmnent before resuspension. Thus they are not able to sense small chemical uctuations necessary for clustering to occur when evolving in a relatively poor medium. (Bottom) Inuence of the internal processes stiffness. When the individual response function *φ* is not stiff, the effect of dispersion is too strong and no pulse wave propagates, as opposed to Fig. 3. Specific parameters are: *δ* = 10 and *N*_0_ = 10^3^. In mathematical models of bacterial chemotaxis, it is commonly accepted that adaptation of cells to large chemoattractant changes acts through the measurement of relative time variations: *S* ^−1^ *DS*/*Dt*. In our context, this is to say that the stiffness parameter *δ* is proportional to the chemical level *S*. Hence after having dramatically changed the environment and before bacteria adapt themselves, we can consider that the response function *φ* is not stiff.

When the stiffness assumption for the internal response function is relaxed, so that dispersion effects become too strong, no pulse propagation is observed numerically (Fig. 4). This is in agreement with analytical results obtained for the zero-speed solution in the absence of nutriment. Indeed the cluster becomes unstable as 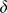 gets large (11).

**Figure 5:**
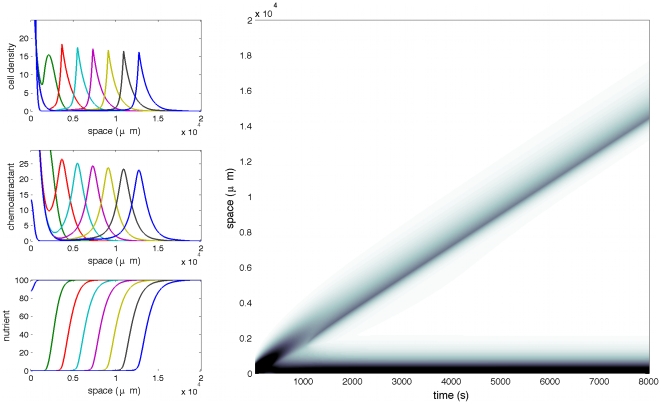
Coexistence of a stationary cluster and a traveling pulse. At low level of nutrient the cell population splits into two subpopulations. A fraction remains trapped at the boundary (as a stationary profile) and a fraction travels accross the channel with constant speed. Specific parameters are: *δ* = 10^−1^ and *N*_0_ = 10^2^.

When the initial level of nutrient is low (or equivalently the consumption rate is high), and conditions for a pulse to travel are fulfilled, then only part of the bacterial population leaves the initial bump (Fig. 5). The solution appears to be the superposition of a traveling pulse and a stationary state (admissible in the absence of nutrient). Solitary modes with smaller amplitudes may appear at the back of the leading one (not shown). To predict which fraction of mass starts traveling turns out to be a difficult question.

## Discussion

We present in this article a simple mathematical description for the collective motion of bacterial pulses with constant speed and asymmetric profile in a channel. The nature of this model significantly differs from the classical Keller-Segel system although it belongs to the same class of drift-diffusion equations. Our model is formally derived from a mesoscopic description of the bacterial density, which allows for a more accurate expression of the cell flux at the macroscopic level.

The main conclusion of our work is the compatibility of the description of individual cell motion at the mesoscopic scale with the macroscopic observations of collective cell movements. The run-and-tumble process is qualitatively and quantitatively consistent with the propagation of a pulse at constant speed.

We do not discuss the conditions ensuring the existence of a traveling pulse solution to the system (5). This has been performed in the case of a stiff response function when computations are tractable analytically. We conjecture that existence holds under rather general assumptions, but such mathematical developments are beyond the scope of this work.

We point out the theoretical connection between the present work and the observations of Mittal *et al.* [38]. The latter corresponds somehow to zero-speed traveling pulses, namely stationary clusters of bacteria. Our approach can be summarized as follows: a nutrient is added to pull chemotactic clusters of cells. This creates an imbalance in the fluxes which induces the asymmetry of the traveling profile.

### Quantitative and qualitative conclusions

We are able to compute the quantitative features of the traveling pulse in the case of a stiff response function. According to (8) the theoretical pulse speed does not depend upon the total number of cells. This can be related to experimental evidence by Mittal *et al.* [38] where bacteria self-organize into size-independent clusters. In the case of a smooth tumbling kernel in (3), our model would predict a dependency of the speed upon the quantity of cells. But this analysis suggests that the number of cells is presumably not a sensitive biophysical parameter.

The speed also does not depend on the effective diffusion coefficient of bacteria when the response function is stiff. Therefore we expect to get the same formula if we follow the hyperbolic approach of [31] in order to derive a macroscopic model. Indeed the main difference is the diffusion coefficient which is very small in the hyperbolic scaling. Nevertheless, the density distribution would be very different, being much more confined when described by the hyperbolic system. Furthermore, scaling back the system to its original variables, we would obtain a pulse speed being comparable to the individual speed of bacteria (Materials and Methods). This is clearly not the case.

The asymmetry factor is another key outcome of the experimental observations. We are able to give a formula for this asymmetry when the response function is stiff. It turns out that asymmetry is favoured when 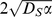 is negligible with respect to the speed of the pulse 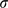 (9). The former parameter is known as the propagation speed of a reaction-diffusion front [12], [40], except that the sign of 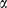 is the opposite.

Although the chemotactic equation of (1)–(2) is significantly different from the standard Keller-Segel model, they coincide in the linear regime. It is well known that the Keller-Segel system is subject to a bifurcation phenomenon due to its quadratic nonlinearity [9], [10]. In the context of cluster formation, we learn from (11) that the stiffness parameter 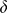 plays an important role in the stability of the homogeneous (flat) state. In other words, it is required that the bacteria are sufficiently sensitive in order to form a stable cluster. Clearly the same kind of mechanism acts here (Fig. 3 as opposed to Fig. 4). However there is no mathematical argumentation to sustain those numerical and intuitive evidence yet.

The influence of the stiffness property of the signal integration process is clear from numerical simulations of the full model (1)–(2). When the response function is smooth, dispersion effects are too strong and the population spreads out (Fig. 4). On the other hand, a stiff response function enables the cells to remain packed under the effect of the self-attractive chemical potential 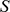. Establishing the exact conditions that guarantee the propagation of a traveling pulse seems to be a challenging task.

Dynamics of the nutrient have no influence when the response function is stiff (only the sign of the gradient is important). However the evolution of the nutrient plays an important role when the response function is not stiff. It may happen than only part of the population starts traveling when the nutrient is initially at a low level (or is consumed with fast rate). A fraction remains trapped on the boundary, in a cluster configuration, while the rest of the population travels independently with constant speed (Fig. 5).

### Perspectives

The next step consists in working at the kinetic level. Much has to be done for the design of efficient numerical methods for the description of collective motion of cells subject to chemotactic interactions. It would also be feasible to point out the dependency of the tumbling operator upon some internal variable (*e.g.* the cytoplasmic concentration of the phosphorylated form of the protein CheY, which is responsible for the reversal of motors). This approach carries out the coupling between an internal protein network and the external chemoattractant signals [39], [41]. Kinetic models are also relevant for describing this microscopic mechanism [30], [42] (the network is basically transported along the cells' trajectories). However the increase in complexity forces to reduce the size of the network, and to use rather caricatural systems mimicking high sensitivity to small temporal variations (excitation) and adaptation to constant levels of the chemoattractant.

Assuming independent integration of the chemical signals constitutes a strong hypothesis of our model. There exist two main membranous receptors triggering chemotaxis, namely Tar and Tsr. As the signals which act in the present experiments are not perfectly determined, we have considered the simplest configuration. To further analyse the interaction between the external signals, one should include more in-depth biological description of the competition for a single class of receptor [43].

## Materials and Methods

### Strain

We use the *E. coli* strain RP437 which is considered wild type for motility and chemotaxis. It is transformed with a pZE1R-gfp maintained by a resistance to ampicillin. The bacteria constitutively express a high level of green fluorescent protein which is necessary for low magnification fluorescence video microscopy.

### Culture

We grow the bacteria with 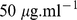 ampicillin on LB agar petri dishes at 37°C and keep them for a maximum of 5 days at 4°C. The “unlimited nutrient” culture medium is M9 supplemented with 4% D-Glucose, 1% Bacto Casamino Acids and 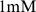 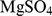. Before each experiment described here, a single colony is inoculated in 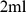 of this medium (and 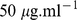 ampicillin) and grown at 30°C under agitation until an OD600 of 0.5 is reached. We use 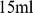 falcon tube with two positions caps to make sure that oxygen is not limited during growth.

### Micro fabrication

The fabrication of micro channels are based on usual soft lithography techniques [44]. 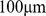 high patterns are micro fabricated on silicon wafers using SU-8 100 resin from MICROCHEM. The PDMS is molded on the wafer and peeled after curing. A clean glass slide and the micro patterned PDMS are plasma treated for 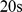 and directly placed in contact thereby forming a PDMS/glass micro channel. The result is a 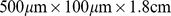 channel (width
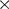
height
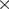
length) that is then filled with the homogeneous suspension of motile bacteria and sealed with a fast curing epoxy resin.

### Centrifugation

The glass silde is gently centrifuged (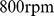, 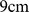 from the axis) at room temperature for 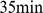. The bacteria accumulate at the end of a channel and stay motile.

### Video microscopy

The channels is then placed in a chamber at constant temperature (30°C) under a Leica MZ16F stereomicroscope equipped for fluorescence. A CCD camera (CoolSnapHQ, Roper Scientific) records one image every 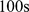 of the fluorescence signal in the channel.

### Image processing

The movie is then processed using Matlab. We detect in each frame the position of the pulse by its maximum and extract its speed by fitting the successive positions by a linear regression.

### Kinetic framework

The classical theory of drift-diffusion limit for kinetic modeling of bacterial chemotaxis is a way to compute the macroscopic fluxes 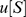, 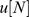 in (2) [27]. Because we assume a linear integration of the different signals for each individual, we restrict the following presentation to the action of a single chemical species 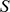.

The population of bacteria can be described at the mesoscopic scale by its local density 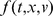 of cells located at the position 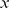 and swimming with velocity 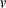. The kinetic equation proposed in the pioneering works of Alt, Dunbar and Othmer [5], [6] combines free runs at speed 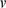, and tumbling events changing velocity from 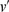 (anterior) to 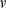 (posterior), according to the Boltzman type equation:
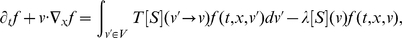
(12)where the tumbling rate satisfies 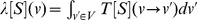. The velocity space 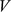 is bounded and symmetric, usually 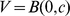 or 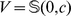 (bacteria having presumably constant speed). As we deal with the idealization of a two-dimensional phenomenon in one dimension of space, we shall perform our computations for 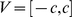, but the results contained in this paper do not depend on this particular choice. Kinetic models of chemotaxis have been studied recently in [42], [45], [46].

**Figure 6:**
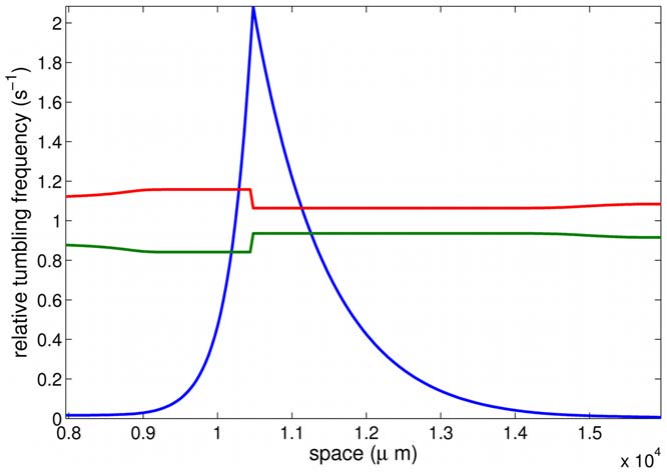
Relative tumbling frequencies (at the mesoscopic scale) obtained from the numerical experiment described in **Fig. 3**: the tumbling probability is higher when moving to the left (upper red line) at the back of the pulse, whereas the tumbling probability when moving to the right is lower (lower green line), resulting in a net ux towards the right, as the pulse travels (see **Fig. 3**). Notice that these two curves are not symmetric w.r.t. to the basal rate 1, but the symmetry defect is of lower order. The peak location is also shown for the sake of completeness (blue line).

The turning kernel 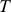 describes the frequency of changing trajectories, from 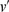 to 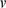. It expresses the way external chemicals may influence cell trajectories. A single bacterium is able to sense time variations of a chemical along its trajectory (through a time convolution whose kernel is well described since the experiments performed by Segall *et al.* [37]). For the sake of simplicity we neglect any memory effect, and we assume that a cell is able of sensing the variation of the chemical concentration along its trajectory. Following [31], this is to say that 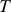 is given by the expression
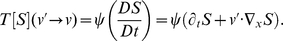
(13)The signal integration function 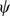 is non-negative and decreasing, expressing that cells are less likely to tumble (thus perform longer runs) when the external chemical signal increases (see Fig. 6 for such a tumbling kernel in the context of the present application). It is expected to have a stiff transition at 0, when the directional time derivative of the signal changes sign [37], [39], [41]. Our study in Section ‘Numerical insights’ boils down to the influence of the stiffness, by introducing a one parameter family of functions 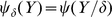.

### Scales

The main parameters of the model are the total number of bacteria 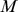 which is conserved, the maximum speed of a single bacterium 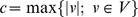, and the mean turning frequency 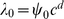 (where 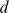 denotes the dimension of space according to our discussion above). The main unknown is the speed of the traveling pulse, denoted by 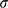. We rescale the kinetic model (12) into a nondimensional form as follows:
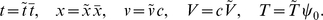
We aim at describing traveling pulses in the regime 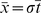. Experimental evidence show that the bulk velocity 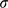 is much lower than the speed of a single bacterium 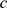. This motivates to introduce the ratio 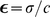. According to experimental measurements, we have 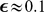. The kinetic equation writes:
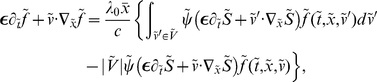
(14)where 
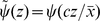
. Following the experimental setting (see Introduction, Fig. 1 and Fig. 2) and the biological knowledge [4], we choose the scales 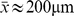, 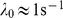, and 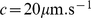. Hence 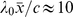. Therefore we rewrite this ratio as:
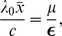
where the nondimensional coefficient 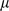 is of order 1.

### Drift-diffusion limit of kinetic models

To perform a drift-diffusion limit when 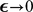 (cf. [9], [27], [28], [32], and [29], [31] for other scaling limits, *e.g.* hyperbolic), we shall assume that the variations of 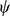 around its meanvalue 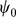 are of amplitude 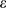 at most. It writes in the nondimensional version as follows: 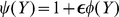. Hence the chemotactic contribution is a perturbation of order 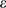 of a unbiased process which is constant in our case because the turning kernel does not depend on the posterior velocity and the first order contribution is required to be symmetric with respect to 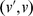. This hypothesis is in agreement with early biological measurements. It is also relevant from the mathematical viewpoint as we are looking for a traveling pulse regime where the speed of the expected pulse is much slower than the speed of a single individual. This argues in favour of a parabolic scaling as performed here.

The resulting macroscopic equation writes as follows, with 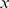 the position along the channel
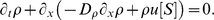
(15)Unlike the classical Keller-Segel model (used for instance by Salman et al. [25]), singularities cannot form (excessively populated aggregates) with the chemotactic flux 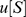 given in (3). This is because the latter remains uniformly bounded (see also Mittal *et al.* [38] where clusters emerge which are plateaus and thus not as singular as described for KS system in a mathematical sense).

We explain in the Text S1 how to derive the parabolic equation from the nondimensional kinetic equation (14). We arrive to equation (15) where the bacterial diffusion coefficient and the chemotactic flux are explicitely given by
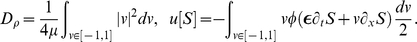
(16)In the limiting case where the internal response function 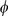 is bivaluated: 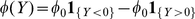, the flux rewrites simply as
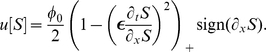
For the sake of comparison, we highlight the corresponding expressions which have been obtained by Dolak and Schmeiser. In [31] authors perform a hyperbolic scaling limit leading to the following chemotactic equation for the density of bacteria
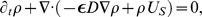
where 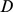 is an anisotropic diffusion tensor and the chemotactic flux is given by
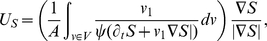
for some renormalizing factor 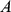. The two approaches do not differ that much at first glance (in particular when 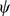 is bivaluated). Notice however that the “small” 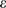 parameter does not appear at the same location: in front of the diffusion coefficient in the hyperbolic limit and inside the chemotactic flux in the parabolic limit.

### Parameter estimation

The macroscopic observable quantities are: the shape of the profile, namely the decay rates 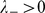 and 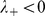, and the pulse speed 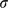. On the other hand, there are three parameters which we were unable to retrieve from the literature: the chemical degradation rate 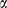 and the effective chemotaxis speeds 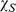 and 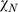 (although [25] indicates 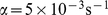 without reference). We deduce from the three constitutive relations (7), (8), the following formulas:
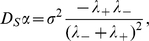


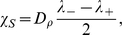


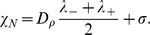
We get from experimental measurements the following values for the observable quantities: 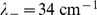, 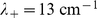 and 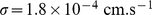.

### Numerical simulations

System (2) is solved using the MATLAB software. The drift-diffusion equation is discretized on a regular grid following a semi-implicit finite-difference scheme. The initial conditions are as follows: a decreasing exponential function centered on the left side of the channel for the cell density, no chemical signal, and a constant level of nutriment 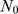. The length of the computational channel is 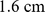.

## Supporting Information

Text S1 The Text S1 consists in three parts. First, we provide analytical details yielding Formulae (8) and (9) in section “Results”. Second, we perform the linear stability analysis referred to in section “Results”. Last, we perform the drift-diffusion limit which yields to equations (15)–(16) in Materials and Methods.
(0.07 MB PDF)
