# Supplementary material for: Mathematical Description of Bacterial Traveling Pulses
Source: PLoS Comput Biol. 2010 Aug 19;6(8):e1000890. doi: 10.1371/journal.pcbi.1000890 (PMC2924242; doi:10.1371/journal.pcbi.1000890)
Supplement: Text S1 — The Text S1 consists in three parts. First, we provide analytical details yielding Formulae (8) and (9) in section “Results”. Second, we perform the linear stability analysis referred to in section “Results”. Last, we perform the drift-diffusion limit which yields to equations (15)–(16) in Materials and Methods. (0.07 MB PDF) [file pcbi.1000890.s001.pdf]

Supporting Information "Text S1"

Computation of the speed of the pulse

We provide below analytical details yielding Formulae (8) and (9) in Section "Results".

Assuming that the sign of the chemical gradient switches at $z = 0$, the cell density of the traveling pulse is given by

$$\rho(z) = \begin{cases} \rho_0 \exp\left(\lambda^- z\right) , & \lambda^- = \dfrac{-\sigma + (\chi_S + \chi_N)}{D_\rho} > 0 , \qquad \text{if } z < 0 , \\ \rho_0 \exp\left(\lambda^+ z\right) , & \lambda^+ = \dfrac{-\sigma + (-\chi_S + \chi_N)}{D_\rho} < 0 , \qquad \text{if } z > 0 . \end{cases}$$

The chemotactic field is given by $S(z) = (K * \beta\rho)(z)$, where the fundamental solution of the diffusion equation for $S(z)$ is

$$K(z) = a_1 \exp(-a_2|z| - a_3 z) , \quad a_1 = \frac{1}{2a_2 D_S} , \quad a_2 = \sqrt{a_3^2 + \frac{\alpha}{D_S}} , \quad a_3 = \frac{\sigma}{2D_S} .$$

To match the transition in monotonicity condition, the chemical signal should satisfy $S'(0) = 0$, that is $(K' * \beta\rho)(0) = 0$, which leads to

$$\begin{aligned} 0 &= \rho_0 \int_{-\infty}^{0} a_1(a_2 + a_3) \exp(a_2 z + a_3 z) \exp(\lambda^- z)\, dz \\ &\quad + \rho_0 \int_{0}^{\infty} a_1(-a_2 + a_3) \exp(-a_2 z + a_3 z) \exp(\lambda^+ z)\, dz \\ 0 &= a_1 \left(\frac{a_2 + a_3}{a_2 + a_3 + \lambda^-} - \frac{-a_2 + a_3}{-a_2 + a_3 + \lambda^+} \right) . \end{aligned}$$

This leads to the following equation that we shall invert to obtain the front speed:

$$\begin{aligned} \frac{\lambda^-}{|\lambda^+|} &= \frac{a_2 + a_3}{a_2 - a_3} , \\ \frac{-\sigma + (\chi_S + \chi_N)}{\sigma + (\chi_S - \chi_N)} &= \frac{\sqrt{4D_S\alpha + \sigma^2} + \sigma}{\sqrt{4D_S\alpha + \sigma^2} - \sigma} . \end{aligned}$$

Dispersion relation for the cluster formation issue

We perform below the linear stability analysis refered to in Section "Results".

Consider the following simple model for cluster formation, in the absence of an external nutrient N.

$$\begin{cases} \partial_t \rho = D_\rho \partial_{xx}^2 \rho - \partial_x \left(\rho u[S]\right) , \quad u[S] = -\displaystyle\int_{v\in(-c,c)} v\phi(v\partial_x S) \frac{dv}{|V|} , \\ -D_S \partial_{xx}^2 S + \alpha S = \beta\rho . \end{cases} \tag{1}$$

We rewrite $\alpha/D_S = l^{-2}$,where l denotes the range of action of the chemical signal. We investigate the linear stability of the stationary state $(\overline{\rho}, \overline{S}) = (\rho_0, \beta\alpha^{-1}\rho_0)$ where ρ_0 denotes the (constant) reference density over the domain $[0, L]$.

We introduce the deviation to the stationary state: $\tilde{\rho} = \rho - \overline{\rho}$, $\tilde{S} = S - \overline{S}$. Then the linearized system writes close to $(\overline{\rho}, \overline{S})$:

$$\begin{cases} \partial_t \tilde{\rho} = D_\rho \partial_{xx}^2 \tilde{\rho} - \partial_x \left(\rho_0 \widetilde{u[S]}\right) , \quad \widetilde{u[S]} = -\displaystyle\int_{v\in(-c,c)} v^2 \phi'(0) \partial_x \tilde{S} \frac{dv}{|V|} = \chi_S \langle v^2 \rangle \frac{\partial_x \tilde{S}}{\delta} , \\ -D_S \partial_{xx}^2 \tilde{S} + \alpha \tilde{S} = \beta\tilde{\rho} . \end{cases} \tag{2}$$

We have introduced our stiffness parameter $\delta = -\phi'(0)^{-1}$. The associated eigenvalue problem reduces to the following dispersion relation for $\xi = 2\pi k/L$,

$$\lambda(k) = -D_\rho \xi^2 + \frac{\rho_0 \langle v^2 \rangle}{\delta} \frac{\beta \xi^2}{\alpha + D_S \xi^2} \, .$$

Due to the conservation of mass, we shall only consider $k \geq 1$. The eigenvalue remains negative for any frequency if the following inequality is fulfilled:

$$\frac{\beta \rho_0 \langle v^2 \rangle}{\delta} < \alpha D_\rho + D_\rho D_S \xi^2 \, , \quad \text{or equivalently} \quad \rho_0 l < \delta \frac{D_\rho D_S}{\beta l \langle v^2 \rangle} \left(1 + (2\pi)^2 \left(\frac{l}{L} \right)^2 \right) . \tag{3}$$

Derivation of the macroscopic model from the kinetic equation

We perform below the drift-diffusion limit which yields to equations (15)-(16) in Materials and Methods.

We start from the nondimensional kinetic equation

$$\epsilon \partial_t f + v \cdot \nabla_x f = \frac{\mu}{\epsilon} \left\{ \int_{v' \in V} \left(1 + \epsilon \phi[S](v')\right) f(t,x,v') \, dv' \; - |V| \left(1 + \epsilon \phi[S](v)\right) f(t,x,v) \right\} ,$$

which reads as follows,

$$\epsilon \partial_t f + v \cdot \nabla_x f = \frac{\mu}{\epsilon} \left(\rho(t,x) - |V| f(t,x,v) \right) + \mu \left(\int_{v' \in V} \phi[S](v') f(t,x,v') \, dv' - |V| \phi[S](v) f(t,x,v) \right) . \tag{4}$$

Therefore the dominant contribution in the tumbling operator is a relaxation towards a uniform distribution in velocity at each position: $f(t,x,v) = \rho(t,x) F(v)$ as $\epsilon \to 0$, where $F(v) = |V|^{-1} \mathbf{1}_{\{v \in V\}}$. Notice that more involved velocity profiles can be handled [1, 2], but this is irrelevant in our setting as the tumbling frequency does not depend on the posterior velocity v.

The space density $\rho(t,x)$ remains to be determined. For this purpose we first integrate with respect to velocity v and we obtain the equation of motion for the local density $\rho(t,x) = \int_{v \in V} f(t,x,v) dv$:

$$\partial_t \rho + \nabla \cdot j = 0 \, , \quad j = \epsilon^{-1} \int_{v \in V} v f(t,x,v) \, dv \, .$$

To determine the bacterial flow j we integrate (4) against v:

$$\epsilon \partial_t \left(\int_{v \in V} v f(t,x,v) \, dv \right) + \nabla_x \cdot \left(\int_{v \in V} v \otimes v f(t,x,v) \, dv \right) = -\mu |V| j - \mu |V| \int_{v \in V} v \phi[S](v) f(t,x,v) \, dv \, .$$

We obtain formally, as $\epsilon \to 0$:

$$j = -\nabla_x \left(\rho(t,x) \frac{1}{\mu d |V|^2} \int_{v \in V} |v|^2 \, dv \right) - \rho(t,x) \frac{1}{|V|} \int_{v \in V} v \phi[S](v) \, dv \, . \tag{5}$$

Finally, the drift-diffusion limit equation reads in one dimension of space:

$$\partial_t \rho = \left(\frac{1}{4\mu} \int_{v \in [-1,1]} |v|^2 \, dv \right) \partial^2_{xx} \rho + \partial_x \left(\rho \int_{v \in [-1,1]} v \phi \left(\epsilon \partial_t S + v \partial_x S \right) \frac{dv}{2} \right) . \tag{6}$$

To sum up, we have derived a macroscopic drift-diffusion equation, where the bacterial diffusion coefficient and the chemotactic flux are given by:

$$D_\rho = \frac{1}{4\mu}\int_{v\in[-1,1]} |v|^2\, dv\,, \quad u[S] = -\int_{v\in[-1,1]} v\phi\left(\epsilon\partial_t S + v\partial_x S\right)\frac{dv}{2}\,. \tag{7}$$

In the limiting case where the internal response function ϕ is bivaluated: $\phi(Y) = \phi_0\mathbf{1}_{\{Y<0\}} - \phi_0\mathbf{1}_{\{Y>0\}}$, the flux rewrites simply as

$$u[S] = \frac{\phi_0}{2}\left(1 - \left(\epsilon\frac{\partial_t S}{\partial_x S}\right)^2\right)_+ \operatorname{sign}\left(\partial_x S\right).$$

For simplicity we set $\epsilon = 0$ in the main text. This does not change the qualitative and quantitative results for the degree of precision we are looking for.

References

1. Chalub F, Markowich P, Perthame B, Schmeiser C (2004) Kinetic models for chemotaxis and their drift-diffusion limits. Monatsh Math 142: 123–141.

2. Perthame B (2004) PDE models for chemotactic movements: parabolic, hyperbolic and kinetic. Appl Math 49: 539–564.
